# Acceptability of the 8-case objective structured clinical examination of medical students in Korea using generalizability theory: a reliability study

**DOI:** 10.3352/jeehp.2022.19.26

**Published:** 2022-09-08

**Authors:** Song Yi Park, Sang-Hwa Lee, Min-Jeong Kim, Ki-Hwan Ji, Ji Ho Ryu

**Affiliations:** 1 Department of Emergency Medicine, Dong-A University College of Medicine, Busan, Korea; 2 Department of Medical Education, Dong-A University College of Medicine, Busan, Korea; 3 Department of Medical Education and Neurology, Kosin University College of Medicine, Busan, Korea; 4 Department of Neurology, Busan Paik Hospital, College of Medicine, Inje University, Busan, Korea; 5 Department of Emergency Medicine, Pusan National University School of Medicine, Busan, Korea; Hallym University, Korea

**Keywords:** Medical students, Educational measurement, Reproducibility of results, Republic of Korea, Psychometrics

## Abstract

**Purpose:**

This study investigated whether the reliability was acceptable when the number of cases in the objective structured clinical examination (OSCE) decreased from 12 to 8 using generalizability theory (GT).

**Methods:**

This psychometric study analyzed the OSCE data of 439 fourth-year medical students conducted in the Busan and Gyeongnam areas of South Korea from July 12 to 15, 2021. The generalizability study (G-study) considered 3 facets—students (p), cases (c), and items (i)—and designed the analysis as p×(i:c) due to items being nested in a case. The acceptable generalizability (G) coefficient was set to 0.70. The G-study and decision study (D-study) were performed using G String IV ver. 6.3.8 (Papawork, Hamilton, ON, Canada).

**Results:**

All G coefficients except for July 14 (0.69) were above 0.70. The major sources of variance components (VCs) were items nested in cases (i:c), from 51.34% to 57.70%, and residual error (pi:c), from 39.55% to 43.26%. The proportion of VCs in cases was negligible, ranging from 0% to 2.03%.

**Conclusion:**

The case numbers decreased in the 2021 Busan and Gyeongnam OSCE. However, the reliability was acceptable. In the D-study, reliability was maintained at 0.70 or higher if there were more than 21 items/case in 8 cases and more than 18 items/case in 9 cases. However, according to the G-study, increasing the number of items nested in cases rather than the number of cases could further improve reliability. The consortium needs to maintain a case bank with various items to implement a reliable blueprinting combination for the OSCE.

## Introduction

### Background/rationale

Test reliability refers to the degree to which a test is consistent and stable in measuring what it is intended to measure [1]. Reliability is a central concept in test theory, as examiners and examinees want a test that gives similar results on different occasions. Test theory has 2 approaches to measuring reliability classical test theory (CTT) and generalizability theory (GT).

CTT usually works well with multiple-choice tests, in which all examinees answer identical questions. However, CTT does not work well with clinical skill examinations in which students do not see the same patients, and the students are not evaluated by the same examiners simultaneously. Thus, the scores of examinees contain variations according to the examiners and clinical scenarios. These variations are a potential source of measurement error [2].

In GT, sources of variation are referred to as facets. These may include persons (students/examinees), raters (examiners), items, cases, and station settings. GT answers how similar the examinee’s score will be in the different tests and scenarios. Specifically, this theory can answer the question of whether this result could be generalized with more stations and fewer examiners in the new objective structured clinical examination (OSCE). The purpose of GT is to quantify the components of the error caused by each facet and the interaction of facets. GT analysis comprises 2 stages: a generalizability study (G-study) and a decision study (D-study). In the G-study, variance components (VCs) from the facets are estimated, and the reliability is calculated. There are 2 reliability indices: generalizability coefficients (G coefficients), which incorporate relative error variance and are used for normative assessments, and Phi coefficients, which contain absolute error variance and are used for criterion-based assessments. After G-study, using the VCs, a post hoc projection of reliability is examined through the D-study. By applying a simulated D-study, it would be possible to investigate how the G coefficients would change under a different examination setting and consequently determine theoretically reliable settings of a clinical examination [3].

Therefore, GT is more informative than CTT for measuring the reliability of clinical examinations [4]. If the form of the OSCE has been changed, a reliability analysis must be performed subsequently, and VC should be analyzed.

### Objectives

The research question of this study was whether the reliability was acceptable when the number of cases in the OSCE decreased from 12 to 8. This study aimed to examine the reliability of medical school OSCEs conducted in South Korea using GT.

## Methods

### Ethics statement

Since this study was not about human subjects or human-originated materials, informed consent from subjects was not indicated and waived. The Institutional Review Board of Dong-A University approved this study protocol (IRB approval no., 2-1040709-AB-N-01-202206-HR-031-02).

### Study design

This was an explorative study to model the implementation of GT. Specifically, this was a psychometric study aimed at measuring the reliability of the OSCE. The present study analyzed clinical skill examination data from 439 fourth-year medical students in the Busan and Gyeongnam areas of South Korea from July 12 to 15, 2021.

### Setting

There are 5 medical schools in the Busan and Gyeongnam areas, located in the southeastern part of South Korea. These 5 medical schools form the Busan-Gyeongnam Clinical Skill Examination (BGCSE) consortium. Since 2014, the consortium has conducted joint clinical skill examinations annually as normative evaluations for third- and fourth-year medical students.

In the 2021 BGCSE, there was a change in the form of the OSCE due to changes in the Korean Medical Licensing Examination (KMLE) by the Korea Health Personnel Licensing Examination Institute. In 2022, the number of OSCE simulations of the KMLE was scheduled to be reduced from 12 to 10. However, the BGCSE consortium lacked the resources to operate all 10 simulations, which required a further reduction to 8. As a result, the OSCE comprised 7 stations where students encountered standardized patients (SPs) and 1 station where students performed procedures on a manikin. Table 1 shows the number of examinees, the topics of the cases, and the number of items in the cases on each OSCE day. The average number of items per case on each OSCE day was 20. Students were given 12 minutes at each station.

By 2020, the consortium had tracked the reliability of the OSCE using Cronbach’s α, and it remained at an acceptable level (above 0.70). However, with the change in the 2021 OSCE, it was necessary to identify the reliability of the test and its error components. Consequently, the consortium decided to analyze the reliability using GT.

The examiners’ training proceeded in the same way as usual. Physician examiners from 4 medical schools evaluated examinees’ performance at each station by completing the checklist and assigning a value from global rating scales. The SPs’ training also proceeded in the same way as usual. The experienced SP trainer trained SPs on scenarios for 2 hours, and they rehearsed for more than 2 hours. All SPs had more than 5 years of SP experience with the BGCSE consortium.

### Participants

A total of 439 fourth-year medical students from 5 medical schools participated in the BGCSE at 4 medical school skill simulation centers for 4 days, from July 12 to 15, 2021.

### Variables

In OSCEs, examples of facets usually include students (p), cases (c), items (i), and raters (r), among others. GT estimates the variance associated with each facet and provides information about the examination’s measurement characteristics. For example, students (p) refer to the variability in scores between examinees that reflects the true difference in competency between students. A greater variance between students indicates that the difference is due to examinee competency, not measurement errors. Cases (c) refer to the variability in difficulty associated with SP encounters in the OSCE. In this study, examinees were randomly assigned to 8 of 23 cases. Items (i) refer to the variability in difficulty associated with checklist items within each case. Raters (r) refer to the variability among examiners. In this study, only 1 rater assessed each case. Thus, there was no variability caused by different raters. In the OSCE, there are interactions between facets. For instance, person-by-case (p×c) interactions indicate differences in student performance according to the cases. The proportion of VCs from each facet provides valuable information about the examination, such as whether the test discriminates high-performance students from low-performance students and whether the number of cases and items is sufficient for reliability.

In this study, we defined 3 facets—students (p), cases (c), and items (i)—and designed them as p×(i:c) due to items being nested in a case. Five types of VCs can be derived from this design: (1) p, (2) c, (3) i:c, (4) p×c, and (5) p×(i:c).

### Study outcomes

We set the primary outcomes as examining the reliability presented as G coefficients and analyzing the VCs on each OSCE examination day (G-study). We set the acceptable reliability level of G coefficients to 0.70 [5]. Since this examination was a normative evaluation, phi coefficient criteria were not set. We set the secondary outcomes as the D-study. Using estimates of VCs via the G-study, a post hoc projection of reliability was examined.

### Data sources/measurement

The data analyzed in this study were from the BGCSE consortium. The scores of examinees’ clinical performance were inserted by faculty examiners using a computer program, and the results were automatically processed. All data were recorded in an Excel spreadsheet (Microsoft Corp., Redmond, WA, USA) and available at Dataset 1.

### Bias

No bias was found in the study scheme.

### Study size

A sample size was not calculated due to the nature of the study design.

### Statistical methods

Descriptive statistics for OSCE scores were calculated, including the mean and standard deviation of each case. The G-study and D-study were performed using G String IV ver. 6.3.8 (2013; Papaworx, Hamilton, ON, Canada). G String IV is a user-centered Windows program that applies GT to analyze empirical datasets. It uses Brennan’s urGenova command-line program to perform the analogous analysis of variance procedure necessary to estimate VCs. It was designed and coded by Ralph Bloch at Papaworx as part of a project commissioned by the Medical Council of Canada. In 2018, G String V was released, and G String can be downloaded for free from the website papaworx.com.

## Results

### Participants

A total of 439 medical students completed the BGCSE, and 128 faculty members participated as examiners.

### Main results

The descriptive statistics are shown in Table 2. Raw score data of examinees for each OSCE day are available at Dataset 1.

#### Generalizability study

All G coefficients except that for July 14 were above 0.70. Items nested in cases (i:c) and residual errors [p×(i:c)] were the major sources of VCs on all examination days (Table 3).

#### Decision study

Table 4 shows the number of items that reached acceptable G coefficients according to the number of cases. As the number of cases increased, the number of items that met the reliability decreased. In 10 cases, the number of items to secure reliability was 18 for all OSCE days. However, there were 21 items in 8 cases.

## Discussion

### Key results

In the 2021 BGCSE, when the number of cases changed from 12 to 8, the G coefficient was at an acceptable level (above 0.70) except for 1 of the 4 examination days. Most VCs were attributed to the items nested in the case and residual error. If the stakes of the OSCE are changed and the reliability needs to be increased, increasing the number of items nested in each case rather than the number of cases would be reasonable.

### Interpretation

According to a systematic review regarding real-world OSCE reliability, the overall reliability presented as α coefficients in medical school examinations was 0.66 (95% confidence interval, 0.62–0.70), which was below the generally accepted minimum reliability [6]. However, the reliability coefficients seem to depend on the purpose of the assessment. If the stakes are high, such as certification, professionals suggest a reliability of at least 0.90. However, for moderate-stakes assessments such as summative examinations in medical school, the reliability is expected to range from 0.80 to 0.89. Lower-stakes assessments, such as formative assessments or those administered by local faculty, would be expected to range from 0.70 to 0.79 [5]. The stakes of the BGCSE are considered low to moderate, as a formative assessment.

According to the D-study, there are 2 approaches for G coefficients above 0.70. One is increasing the number of cases from 8 to 9 or 10, and the other is increasing the number of items nested in cases to more than 20 while maintaining the number of cases at 8.

Each approach has its advantages and disadvantages. Increasing the number of cases will increase reliability, but more resources are needed. If the number of cases rises to 10, the consortium must prepare 2 more cases. This means that an additional 32 physician examiners and 8 SPs will be needed. More staff for the operation of the OSCE and item developers for new cases will also need to join. More manikins and equipment for added stations will also be required. In this case, the consortium will have to consider the cost-effectiveness of the OSCE.

Increasing the number of items will also increase reliability. However, when developing cases, the number of items tends to depend on the case’s topic. For example, as shown in Table 1, the vaccination counseling case (a 32-year-old woman is counseled about vaccination for her 9-month-old baby) included 22 items since many key questions are to be asked before vaccination, such as previous vaccination history and allergy reaction history, and current medication history. However, in the case of intimate partner violence (a 41-year-old woman with a swollen and bruised right eye), there may be fewer key questions. If we add superfluous items, these will have low assessment value and eventually reduce the validity of the case. Thus, it will not always be possible to increase the number of items to secure reliability.

### Comparison with previous studies

It is well known that the major threat to reliable measurements in evaluating performance is case specificity [7]. Case specificity can be defined as a phenomenon in which student performance varies depending on the scenario [8]. This is because some students may have more prior knowledge or experience in some scenarios than others. Previous studies have shown that case specificity in multicase examinations is naturally a significant VC. Therefore, a reliable test is needed for many cases [9,10]. However, recent studies have shown that the number of cases is not necessarily the source of variance. Instead, the source of significant variance can be attributed to items nested in cases or other factors [11,12]. The findings of our study are consistent with recent studies because the proportion of VCs for cases was negligible, from 0.00% to 2.03% (Table 3). Therefore, in this examination, increasing the item number per case can increase the reliability of the examination, since most of the VCs were from items nested in cases (i:c).

This study found that if the OSCE was performed in 8 cases, the G coefficient was above 0.70 when the average number of items was above 21. This means that if the number of items in some cases is more than 21, the number of items in other cases could be less than 21. In this situation, a combination of cases with various items may be important in the blueprinting of the OSCE. The consortium should have sufficient cases in which various items are included in the case bank.

### Limitations

This study has some limitations. First, it was conducted by 1 consortium, although 5 medical schools participated. Applying the same OSCE will result in different findings depending on the student population. Second, items evaluating patient-physician interactions (PPIs) were excluded from the G-study. Because the number and contents of items evaluating PPIs are already set in all cases by the Korea Health Personnel Licensing Examination Institute, the consortium cannot modify them. Third, the items of the cases belong to categories such as history taking, physical examination, and patient education. The composition ratio of these categories may vary depending on the case. For each case, a sub-design using the p×(i:c) structure was possible. However, we did not analyze whether the number of items in the categories was appropriate because it was beyond our research question. Other studies on this topic should be conducted in the future.

### Generalizability

Reliability analysis using GT can improve the reliability of other OSCEs.

### Suggestions

There was 1 examiner for each case in this study, and the rater (r) was not considered in the G-study design. However, we did not verify intrarater reliability. Further research is needed on this topic in the future.

### Conclusion

In the 2021 BGCSE, the case number decreased from 12 to 8. However, the reliability was acceptable. In the D-study, reliability was maintained at 0.70 or higher if there were more than 21 items per case with 8 cases and more than 18 items per case with 9 cases. However, according to the G-study, increasing the number of items nested in cases rather than the number of cases could further improve reliability because most VCs were from items nested in cases. The consortium needs to maintain a case bank with a diverse number of items to implement reliable blueprinting for the OSCE.

## Figures and Tables

**Table 1.** The topic of the cases, the number of examinees, and items of cases on each OSCE day

Date of the OSCE	July 12	July 13	July 14	July 15
The topic of the cases (no. of items)				
Case 1	A 60-year-old man with shortness of breath (19)	A 60-year-old man with shortness of breath (19)	A 32-year-old man with palpitations (19)	A 60-year-old man with shortness of breath (19)
Case 2	A 32-year-old man with diarrhea (18)	A 49-year-old woman with yellowish eyes (23)	A 56-year-old man with blood in his stool (19)	A 39-year-old man with loss of consciousness (17)
Case 3	A 45-year-old woman with hand tremors (19)	A 24-year-old woman with breast pain (16)	A 45-year-old woman with hand tremors (19)	A 46-year-old woman with dizziness (19)
Case 4	A 41-year-old woman with a swollen and bruised right eye (17)	A 50-year-old man counseled for drinking (20)	A 37-year-old man with a backache (21)	A 41-year-old woman with a swollen and bruised right eye (17)
Case 5	A 32-year-old woman for vaccination counseling for her 9-month-old baby (22)	A 32-year-old man with palpitations (19)	A 32-year-old woman for vaccination counseling for her 9-month-old baby (22)	A 51-year-old woman with lower abdominal pain (18)
Case 6	A 43-year-old man with knee pain (20)	A 40-year-old woman with a blood spot in her underwear (17)	A 24-year-old woman with breast pain (16)	A 40-year-old woman with a blood spot in her underwear (17)
Case 7	A 41-year-old woman with memory loss (18)	A 41-year-old woman with memory loss (18)	A 41-year-old woman with memory loss (18)	A 43-year-old man with knee pain (20)
Case 8	A 25-year-old woman with a left arm laceration (wound care) (30)	A 25-year-old man with shortness of breath (arterial blood sampling) (31)	A 25-year-old man diagnosed with pneumonia (blood culture) (26)	A 57-year-old man who suddenly collapsed (resuscitation) (29)

OSCE, objective structured clinical examination.

**Table 2.** Descriptive statistics for OSCE scores

	Date of the OSCE			
	July 12	July 13	July 14	July 15
No. of examinees	111	109	112	107
Case 1				
Max	87.44	96.92	84.17	92.48
Min	35.91	44.41	36.11	45.03
Mean±SD	66.55±10.15	71.40±10.41	68.23±9.89	72.45±9.88
Case 2				
Max	94.62	85.83	93.49	91.11
Min	46.79	33.33	43.65	32.22
Mean±SD	73.82±9.48	68.31±10.38	73.39±8.94	64.73±14.27
Case 3				
Max	94.44	95.56	94.60	85.06
Min	41.92	38.61	42.07	26.67
Mean±SD	70.17±12.17	67.45±11.37	75.13±8.72	53.78±13.43
Case 4				
Max	92.50	85.33	91.11	97.33
Min	15.50	39.33	39.67	41.75
Mean±SD	61.01±13.49	68.58±8.93	66.05±11.07	70.32±10.60
Case 5				
Max	87.69	88.89	90.77	93.33
Min	6.00	38.33	37.96	37.44
Mean±SD	57.52±14.14	68.86±10.83	65.87±11.34	72.24±11.37
Case 6				
Max	82.36	82.94	90.00	93.33
Min	39.48	38.25	32.64	40.00
Mean±SD	59.12±9.29	65.58±9.44	70.88±11.34	59.80±11.35
Case 7				
Max	90.83	100.00	93.33	93.33
Min	34.03	39.72	37.22	39.89
Mean±SD	59.71±12.54	64.47±11.46	62.10±11.67	62.33±9.91
Case 8				
Max	100.00	100.00	100.00	100.00
Min	27.27	33.33	32.14	31.03
Mean±SD	84.79±11.91	80.18±12.89	79.85±15.14	77.41±12.87
Overall (800)^a)^				
Max	662.17	667.30	681.47	669.24
Min	330.01	409.68	348.24	408.54
Mean±SD	532.70±58.00	554.82±48.96	561.51±52.44	533.05±56.52

The score of each case was converted to 100 points. OSCE, objective structured clinical examination; SD, standard deviation.

a) The difference in overall score among the 4 groups was statistically significant (P<0.001) by analysis of variance with the Scheffe post hoc test.

**Table 3.** The generalizability study

Variable	Effect					
	df	T	SS	MS	VC	VC (%)
July 12						
p	110	181.43	181.43	1.65	0.01	1.45
c	7	357.93	357.93	51.13	0.01	2.03
i:c	155	4,748.96	4,391.03	28.33	0.25	51.34
p×c	770	901.36	361.99	0.47	0.01	2.60
p×(i:c)	17,050	8,874.33	3,581.95	0.21	0.21	42.58
G coefficient						0.71
Phi coefficients						0.56
July 13						
p	108	145.29	145.29	1.35	0.01	1.05
c	7	43.21	43.21	6.17	-0.01	0.00
i:c	155	5,374.77	5,331.57	34.4	0.31	57.70
p×c	756	491.79	303.30	0.40	0.01	1.69
p×(i:c)	1,6740	9,421.67	3,598.31	0.21	0.21	39.55
G coefficient						0.70
Phi coefficients						0.56
July 14						
p	111	177.7	177.70	1.60	0.01	1.32
c	7	203.78	203.78	29.11	-0.00	0.00
i:c	152	4,975.13	4,771.35	31.39	0.28	52.94
p×c	777	760.21	378.73	0.49	0.01	2.48
p×(i:c)	16,872	9,367.83	3,836.27	0.23	0.23	43.26
G coefficient						0.69
Phi coefficients						0.59
July 15						
p	106	161.63	161.63	1.52	0.01	1.32
c	7	140.27	140.27	20.03	-0.01	0.00
i:c	148	4,720.79	4,580.52	30.95	0.29	55.99
p×c	742	642.86	340.96	0.46	0.01	2.55
p×(i:c)	15,688	8,455.07	3,231.69	0.21	0.21	40.14
G coefficient						0.70
Phi coefficients						0.59

The variables of the effect are as follows: student (p), case (c), and item (i). The model of p×(i:c) was used in G-string IV ver. 6.3.8 (Papaworx, Hamilton, ON, Canada). df, degrees of freedom; SS, sum of squares; MS, mean square; VC, variance components.

**Table 4.** The decision study

	No. of items													
	15	18	19	20	21	22	23	24	25	26	27	28	29	30
July 12														
No. of case														
8		0.70^a)^	0.71	**0.71**	0.72	0.72	0.72	0.73	0.73	0.73	0.74	0.74	0.74	0.74
		0.54	0.55	**0.56**	0.56	0.57	0.57	0.58	0.58	0.59	0.59	0.59	0.6	0.6
9		0.73^a)^	0.73	0.73	0.74	0.74	0.75	0.75	0.75	0.76	0.76	0.76	0.76	0.77
		0.57	0.58	0.58	0.59	0.60	0.60	0.61	0.61	0.61	0.62	0.62	0.62	0.63
10		0.75^a)^	0.75	0.75	0.76	0.76	0.77	0.77	0.77	0.77	0.78	0.78	0.78	0.78
		0.60	0.60	0.61	0.62	0.62	0.63	0.63	0.63	0.64	0.64	0.65	0.65	0.65
July 13														
No. of case														
8		0.68	0.69	**0.70** ^a)^	0.70	0.71	0.71	0.72	0.72	0.72	0.73	0.73	0.73	0.74
		0.54	0.55	**0.56**	0.57	0.58	0.59	0.60	0.60	0.61	0.61	0.62	0.63	0.63
9		0.71^a)^	0.72	0.72	0.73	0.73	0.74	0.74	0.74	0.75	0.75	0.75	0.76	0.76
		0.57	0.58	0.59	0.60	0.61	0.62	0.62	0.63	0.64	0.64	0.65	0.65	0.66
10		0.73^a)^	0.74	0.74	0.75	0.75	0.76	0.76	0.76	0.77	0.77	0.77	0.78	0.78
		0.60	0.61	0.62	0.63	0.63	0.64	0.65	0.65	0.66	0.67	0.67	0.68	0.68
July 14														
No. of case														
8	0.66	0.68	0.69	**0.69**	0.70^a)^	0.70	0.71	0.71	0.71	0.72	0.72	0.72	0.73	0.73
	0.54	0.57	0.58	**0.59**	0.60	0.61	0.61	0.62	0.63	0.63	0.64	0.64	0.65	0.65
9	0.69	0.71^a)^	0.71	0.72	0.72	0.73	0.73	0.73	0.74	0.74	0.74	0.75	0.75	0.75
	0.57	0.60	0.61	0.62	0.63	0.63	0.64	0.65	0.65	0.66	0.66	0.67	0.67	0.68
10	0.71^a)^	0.73	0.74	0.74	0.74	0.75	0.75	0.76	0.76	0.76	0.76	0.77	0.77	0.77
	0.60	0.63	0.64	0.64	0.65	0.66	0.66	0.67	0.68	0.68	0.69	0.69	0.69	0.70
July 15														
No. of case														
8	0.67	0.69	0.69	**0.70** ^a)^	0.70	0.71	0.71	0.71	0.72	0.72	0.72	0.73	0.73	0.73
	0.54	0.57	0.58	**0.59**	0.60	0.60	0.61	0.62	0.62	0.63	0.63	0.64	0.64	0.65
9	0.69	0.71^a)^	0.72	0.72	0.73	0.73	0.73	0.74	0.74	0.74	0.75	0.75	0.75	0.75
	0.57	0.60	0.61	0.62	0.63	0.63	0.64	0.64	0.65	0.66	0.66	0.67	0.67	0.67
10	0.72^a)^	0.73	0.74	0.74	0.75	0.75	0.75	0.76	0.76	0.76	0.77	0.77	0.77	0.77
	0.60	0.63	0.63	0.64	0.65	0.66	0.66	0.67	0.67	0.68	0.68	0.69	0.69	0.70

Variables are presented as G coefficients (upper) and Phi coefficients (lower). The bold type is the reliability of the OSCE in this study. The acceptable reliability of G coefficients was set to above 0.70. OSCE, objective structured clinical examination.

a) Minimally required item number of that case.

## Footnotes

**Data availability**
Data files are available from Harvard Dataverse: https://doi.org/10.7910/DVN/DJNHCM Dataset 1. Raw score data of examinees for each OSCE.

## Supplementary materials

Supplement 1. Audio recording of the abstract.
